# Meat Quality of the Native Carpathian Goat Breed in Comparison with the Saanen Breed

**DOI:** 10.3390/ani11082220

**Published:** 2021-07-27

**Authors:** Władysław Migdał, Aldona Kawęcka, Jacek Sikora, Łukasz Migdał

**Affiliations:** 1 Department of Animal Product Processing, University of Agriculture in Krakow, ul. Balicka 122, 30-149 Krakow, Poland; wladyslaw.migdal@urk.edu.pl; 2 Department of Sheep and Goat Breeding, National Research Institute of Animal Production, ul. Krakowska 1, 32-083 Balice, Poland; jacek.sikora@iz.edu.pl; 3 Department of Genetics, Animal Breeding and Ethology, University of Agriculture in Krakow, al. Mickiewicza 24/28, 30-059 Krakow, Poland; lukasz.migdal@urk.edu.pl

**Keywords:** male goat kids, Carpathian, Saanen, meat quality

## Abstract

**Simple Summary:**

The aim of this study was to investigate the influence of male goat kid breeds on the basic chemical, fatty and amino acid composition, colour and sensory evaluation of fresh meat. The meat of the Carpathian kids was characterised by a lower content of protein and cholesterol, and a higher content of fat. Despite the higher collagen content, the meat was characterised by lower shear force, less hardness and chewiness, being a more delicate meat compared to the meat from Saanen goats. The meat of native goats was higher concerning the content of phenylalanine, histidine, proline, alanine and tyrosine, as compared to the meat of the Saanen goats. The fat of Carpathian goat meat was characterised by a higher content of monounsaturated acids and a more favourable (lower) saturation index.

**Abstract:**

Goats provide valuable products that are appreciated by consumers who are looking for food that is not only tasty but also healthy, and, probably, one of them is goat meat. Breeding of local breeds such as the native Carpathian goat has been gaining importance in recent years, which creates an opportunity for the development of the goat meat market. The aim of this study was to investigate the influence of goat breed on the basic chemical, fatty and amino acid composition, colour and sensory evaluation of meat. The research material consisted of Carpathian goats from the NRIAP experimental plant located in the southern part of Poland, and goats from a farm keeping Saanen goats in south-eastern Poland. Ten male goat kids from each breed were taken to the NRIAP farm. The quality of meat obtained from the leg (*m. biceps femoris*) of male goat kids about 150 days old at slaughter was analysed. The meat of the Carpathian goat was characterised by a lower content of protein and cholesterol (*p* < 0.01), and a higher content of fat and general collagen compared to the meat from Saanen goats (*p* < 0.05). Cholesterol content in goat meat of both breeds was similar and ranged from 55.08 mg/100 g (Carpathian) to 56.79 mg/100 g (Saanen). Despite the higher collagen content, the goat meat of Carpathian breeds was characterised by lower shear force, less hardness (*p* < 0.05) and chewiness, being a more delicate meat. The fat of Carpathian goat breeds was characterised by a higher content of monounsaturated acids, mainly C 18:1n:9, and a more favourable (lower) saturation index, S/P (*p* < 0.05). The meat of Carpathian goats was characterised by a higher health-promoting quality compared to the meat from Saanen goats. In the goat meat of both breeds, there were no differences between the total content of exogenous and endogenous amino acids. The essential/nonessential amino acids (EAA/NEAA) ratio in the meat of the analysed breeds was 0.88:0.89. However, the meat of the Carpathian goats was statistically significantly higher concerning the content of phenylalanine, histidine, proline, alanine and tyrosine, as compared to the meat of the Saanen goats. The obtained results confirm the high quality of the meat of the local Carpathian breed in comparison to the Saanen breed.

## 1. Introduction

The world population of goats is over 1 billion units. Only during the period from 2000 to 2013 did an important increase in goats occur worldwide (33.79%). Among the continents, Asia constantly holds first place, having a contribution to the total goat population of 59.38% and an increase in the number of goats by 30.23% in the period 2000–2013. Africa comes in second with a contribution of 35% and an increase during the above-mentioned period by 48.61%. In Oceania, a spectacular increase was observed in the number of goats (65.76%) during the same period. In the Americas, the increase was only 3.13%, while in Europe and the E.U. (28), a relative decrease could be noted [1]. In 2017, 12.7 million goats were kept in the European Union, but goat breeding in Europe is very diverse. According to data of EUROSTAT [2] from 2018 reports, it was noted that the majority of goats were kept in Greece (3,625,000), Spain (2,764,000) and, in other European countries outside the EU, 10,634,000 were kept in Turkey. The goat population in Poland has undergone rapid changes - from around 800,000 in 1948, to 40,000 in 1970. Data from 2015 show that it was 58,000 pcs., which was 0.5% of all farm animals [3].

The present goat population in Poland was influenced by the old local general-purpose breeds: Carpathian, Sandomierska and Kazimierzowska. The Sandomierska and Kazimierzowska native breeds are extinct, but efforts at restoring the Carpathian breed were successful and now a small population of these goats is raised in the Sub-Carpathian area [4]. Goats kept in Poland are mostly hybrids obtained from crossing the local population with noble breeds. Of the noble breeds, the meat of the Boer breed plays a dominant role, and, of dairy breeds, the White and Coloured Improved, Saanen and Alpine breeds are dominant [5]. Saanen goats make up approximately 10% of goats entered in the herd books in Poland and their average milk yield is approximately 640 kg [6]. Until recently, in the Polish population, apart from the most common Polish goats, the so-called Polish Improved and Polish Coloured Improved animals with a typical dairy usage, there were local breeds (Kazimierzowska and Carpathian) and non-breed goats—general-purpose type. All-purpose breeds (milk, meat, skins) gradually lost their importance and were replaced by other, one-purpose breeds (dairy or meat) or dual-purpose types (dairy and meat), which was one of the reasons that led to the extinction of the mentioned native goats [7]. 

The tradition of keeping the primitive mountain Carpathian goats and Zackel sheep dates back to the Wallachian colonisation. Beginning from the 12th century, Wallach shepherds (Wallachs were an ethnic group originating from the Balkan Peninsula, living south of the Danube river) started to move with their animal herds (mainly of sheep and goats), which needed pastures, to the north along the Carpathian range, reaching the Oravia and Moravia and to the “Polish” area, the contemporary Beskids [8]. Apart from sheep and cattle, the Wallachs also grazed herds of goats with a lean body structure and a long or semi-long white or coloured coat. In the area of the Polish mountains, so-called Carpathian goats, white mountain goats, could be found. This goat was adapted to the harsh mountain conditions, used fodder very well, was resistant to diseases and had a lean body structure with low-set, strong limbs, a wide and well-arched chest and long and dense coat, allowing animals to survive in difficult conditions. Good milk yield, about 500 kg per year, was enough to feed their offspring and produce Wallachian cheeses [9]. 

The twilight of transhuman pastoralism, a negative campaign regarding goats (the poor people’s animal), goat’s milk and goat products, and the importation of more efficient breeds (Saanen and Toggenburg goats), contributed to the fact that in the second half of the 20th century, this breed was considered extinct. In 2006, actions were taken in an attempt to restore this breed by the National Research Institute of Animal Production Experimental Station in Odrzechowa as part of the statutory activity, using two herds of goats of the Carpathian goat type found in Poland. Two years later, a programme for the conservation of the genetic line of the Carpathian breed of goats was undertaken, and in 2010, by the decision of the Minister of Agriculture and Rural Development, the National Research Institute of Animal Production was recognised as keeping a breeding book for the Carpathian goat. This breed is of versatile utility, with an average milk yield of about 350 kg. Male and female kid goats are used to produce good quality meat [10]. According to Călin et al. [11], Carpathian breed populations still have a high degree of heterogeneity, by reacting positively to improved technologies and through their performance in terms of meat production, characteristics that can be improved and used in terms of economic efficiency. Now, in Poland, Carpathian goats have the largest share in the active population [12].

The aim of the study was to analyse the physicochemical characteristics of the meat of goats from native Carpathian breeds and to compare them with the results of meat quality from goats of the Saanen breed.

## 2. Materials and Methods

### 2.1. Animals and Experimental Design

The experiment was carried out in 2019 on the farm of the National Research Institute of Animal Production (NRIAP), located in the southern part of Poland. The research material consisted of Carpathian goats from a herd of 30 dams from the NRIAP experimental plant located in the southern part of the country, and goats from a farm keeping 30 Saanen goats in south-eastern Poland. All the goats were from twins. Goats on both farms were kept with their mothers until about 60 days of age, where they received crushed oats and hay. After weaning, animals were weighed at 90 days of age and 10 goats from each breed were chosen based on average live weight and were taken to the NRIAP farm. After weaning, they were fed with good quality hay and received 300 g of a concentrated mixture containing: 52% barley, 20% oats, 5% wheat bran, 15% soybean meal, 5% rapeseed expeller and 3% of a mineral mixture. After weaning, the male goats were kept in two group pens (4 × 4 m) by breed. The area of the pens was 1.5 m^2^ per animal. After 10 days of adaptation, the fattening period started, which lasted from day 100 to slaughter on day 150. During this period, they received the same concentrated mixture at the amount of 400 g per animal and hay at will. The animals had constant access to fresh water and licks. After fattening, animals were subjected to slaughter at an EU-licensed abattoir after 24 h feed withdrawal. They were stunned using a captive-bolt pistol. Procedures met the requirements of the Directive 2010/63/EU [13] of the European Parliament and of the Council of 22 September 2010 on the protection of animals used for scientific purposes. After 24 h of chilling at 4 °C, slaughter analysis was performed according to the methodology for small ruminants prepared by the National Research Institute of Animal Production [14]. The carcasses were divided into half-carcasses, and then the right half-carcass was sectioned into cuts. The analysis included post-slaughter carcass evaluation and determination of the proportion of carcass cuts and leg tissue composition. Muscle samples were taken from the leg (*m. biceps femoris*) to determine chemical composition, physicochemical, texture and sensory parameters, as well as the fatty acid profile of the meat. Samples were vacuum packed in polyethylene packs and stored at –20 ◦C for further evaluation.

### 2.2. Meat Physicochemical Properties

Meat colour was determined using a Konica Minolta CM–600d spectrophotometer (Minolta Co., Ltd., Tokyo, Japan) with a 50-mm diameter measuring head in the CIE L*a*b* system, where the L* parameter corresponds to the degree of lightness (L* = 0: black, L* = 100: white), a* and b* are colour components (a* > 0 red, a* < 0 green, b* > 0 yellow, b* < 0 blue). The chromametre was calibrated against a white tile (Y = 93.8, x = 0.3136, y = 0.3192) (CIE, 1986). 

The following items were assessed in the meat samples:

- water content according to the standard (*PN-ISO 1442:2000)* [15],
- fat content according to the standard (*PN-ISO 1444:2000)* [16],
- protein content by Kjeldahl method *(PN-75/A-04018)* [17],
- total ash content according to the standard (*PN-ISO 936:2000)* [18],
- total carbohydrate content assuming that all total solids and water stood for 100%.

The total collagen content was estimated according to Polish Standard *(PN-ISO 3496:2000)* [19]. The absorbance of samples was measured with Novasina spectrophotometer at 558 nm. The hydroxyproline content was read from calibration curve. The total collagen content was calculated from hydroxyproline amount using the coefficient 7.25 and with dilution factors included.

Fatty acid profile was determined by two analytical methods: lipid extraction from meat according to Folch et al. [20] and esterification according to AOAC (1995) [21]. The fatty acid methyl esters were separated by gas chromatography using a TRACE GC ULTRA (Thermo Electron Corporation, Milano, Italy) with a flame ionisation detector (FID) via the SUPELCOWAX 10 column (30 m × 0.25 mm × 0.25 µm). The separation conditions were as follows: helium as the carrier gas, 1 mL/min; FID detector temp. 250 °C; injector temp. 220 °C; oven temp. was maintained at 160 °C and increased (3 °C/min) to 210 °C (35 min); split ratio 10 mL/min. To the obtained fat (approx. 10 mg), 0.5 mL of 0.5 M KOH in methanol was added and heated to 85 °C, after which 1 mL of 12% BF_3_ in methanol was added and reheated at 85 °C. After cooling down to room temperature, 1 mL of hexane and 5 mL of saturated NaCl solution were added. The solution, in the amount of 1 µL, was injected on the chromatograph. Individual fatty acid methyl esters (FAME) were identified by comparison with a standard mixture of 37 FAME components (Supelco, Sigma-Aldrich Co., St. Louis, MO, USA) and CLA isomers (Sigma-Aldrich Co.).

The amino acid composition was determined by means of RP-HPLC using the Waters ACCQ-Tag Ultra Derivatization kit (186003836, Waters, Milford, MA, USA). Using 4 mL of 6 M HCl and 15 μL of phenol at 110 °C for 24 h, 2 mg of the sample were hydrolysed. The sample was sealed in a nitrogen atmosphere during the process of hydrolysis. The acquired hydrolysate was filtered using a syringe filter with a pore diameter of 45 μm and dried under a constant stream of nitrogen. The samples prepared in such a way were diluted accordingly and derivatised by mixing 10 μL of the sample with 70 μL of boron buffer (pH in the range of 8.2–9.0) and 20 μL of 6-aminoquinolyl-N-hydroxysuccinimidylcarbamate (AQC) in a 3 mg concentration of ACQ/mL acetonitrile. The standards were prepared in the same manner as the samples. The separation was carried out using the Dionex Ultimate 3000 HPLC system (Thermo Scientific, Waltham, MA, USA) equipped with an LPG-3400 SD 4-channel gradient pump, WPS 3000 TSL autosampler and VWD 3400RS 4-channel UV/VIS detector. Analysis was performed using a Nova-Pak C18, 4 μm (150 × 3.9 mm) column (Waters, USA). In the elution procedure, an acetate-phosphate buffer (Eluent A) and 60:40 acetonitrile/water (Eluent B) were used according to the procedure recommend by Waters (USA). The separation temperature was set at 37 °C. Detection was carried out at a 240 nm wavelength. Quantitative analysis was performed via 1 point calibration using analytical standards (100 pmol for each concentration).

### 2.3. Texture and Sensory Evaluation

Muscle fragments weighing around 120 g were packed in aluminium foil. Thermal treatment in an electric furnace was carried out at a temperature of 180 ± 2 °C until reaching a muscle temperature of 72 ± 2 °C. The temperature inside the muscles was measured with a digital thermometer using a probe needle. After heat treatment and cooling on ice, cooking loss was determined from meat weight loss, and sensory and texture analyses were performed. 

The texture profile (TPA) of the meat was analysed according to the PN-ISO Norm 11036:1999, with the TA-XT2 texture analyser (Stable Micro Systems Co. LTD., Godalming, UK). Shear force was measured from cylindrical samples (14 mm diameter, 15 mm height), using a Warner–Bratzler attachment (shearing blade thickness of 1.016 mm, V-shaped cutting blade with a 60° angle, corner of the V rounded to a quarter-round of a 2.363 mm diameter circle, spacers providing the gap for the cutting blade to slide of 1.245 mm thickness) and a triangular notch in the blade. Blade speed during the test was 1.5 mm/s. The results are presented as force per area (kg/cm^2^). The meat was roasted to 180 °C with an inner temperature of 78 °C, then cooled to room temperature and the samples were cut out, parallel to the muscle fibres, as cylinders with a diameter of 16 mm and 15 mm in height. The speed of the sampling knife movement during the test was 1.5 mm/s. The results are presented as force working on the surface of the cut (kg/cm^2^). The analysis of texture profile was performed with the above device using a cylindrical sampling probe with a diameter of 50 mm. The test of double pressing the meat samples was conducted up to 70% deformation of their height. The speed of cylinder movements were 2 mm/s, the interval between the 3 pressings was 3 s, whereas the threshold of sample detection was 10 g. TPA parameters were calculated via Texture Exponent software, version 5.1.15.0 (Stable Micro Systems). Texture (hardness, springiness, cohesiveness, chewiness) was analysed using the attached cylinder—50 mm in diameter. The samples were subjected to a double pressing test, applying a force of 10 g to 70% of their height. The cylinder speed was 2 mm/s, and the interval between presses was 3 s.

### 2.4. Sensory Evaluation

Sensory analysis was also performed. After roasting, the meat samples were evaluated using a scaling method according to the Polish Standards: PN-ISO 4121:1998 [22] and PN-ISO 6658:1998 [23]. The following features of the meat were assessed: structure, aroma, tenderness, juiciness and flavour with a 5-point scale [22]. The assessment was made by the 10 panellists consisting of employees from the Department of Animal Product Technology, University of Agriculture in Kraków, trained in goat meat sensory analysis. The panel had years of experience in sensory evaluation practice and were trained theoretically and practically regarding the applied methods [24]. The panellists rinsed their mouths with a sip of water before starting the analysis and between each sample. The meat samples were scored for the analysed characteristics on a scale from 1 (poor—lowest grade) to 5 (excellent—the highest grade) with increments of 0.5. The final scores for different meat features were evaluated as average scores assigned by the tasters. The sensory evaluations were conducted in standard conditions, including room temperature (22 °C), relative humidity 65%, individual booths and white light of approximately 500 lx. Results were analysed in Panel Check v.1.4.2 (PCA; Panel Check V 1.4.2, Nofima, Norway); http://www.panelcheck.com, (accessed on 1 March 2021) using STATIS analysis biplot option.

### 2.5. Statistical Analysis

All samples were obtained at least in duplicates. All results were analysed with t-Student test and presented as mean ± standard deviation. The calculations were performed using Statistica 6.0 [25].

Y_i_ = µ + B_i_ +e_i_

where Y_i_ represents the observations, µ is the overall mean, B_i_ is the effect of breed (i = 1.2), e_i_ represents random error. 

## 3. Results and Their Discussion

The chemical composition and quality of goat meat depends, among others, on the genotype (breed), utility type, sex, age of slaughter, nutrition and breeding system [26,27,28]. In Table 1, the chemical composition of the kid goat meat (non-castrated) slaughtered at the age of five months belonging to two breeds: dairy Saanen and general Carpathian, is presented. The meat of the Carpathian goats was characterised by a lower content of protein and cholesterol, while the content of fat and general collagen was higher compared to the meat of the Saanen goats. According to Horoszewicz and Pieniak-Lendzion [29], white meat from a male goat kid improved breed, slaughtered at 90 days of age, contained 1.29% of fat, while the meat of goats slaughtered on the 150th day contained 2.27% of fat. Borgogno et al. [30] found 3.0 g lipids in 100 g of fresh meat (*longissimus thoracis*) fall chevon (5–5.5 months) kids of Alpine breed. Water/protein ratio ranged from 3.78 (Saanen) to 4.04 (Carpathian), and was similar to the results obtained by Brzostowski et al. [31] for meat of the French Alpine breed (4.18) and French Alpine × Boer goats (3.89). Cholesterol content of the goat meat of both breeds was similar and ranged from 55.08 mg/100 g (Carpathian) to 56.79 mg/100 g (Saanen). By slaughtering 50-day-old French Alpine goats, Brzostowski et al. [31] found lower fat (1.67%) and cholesterol (48.76 mg) content in the meat (quadriceps muscle of the thigh (*m. quadriceps femoris*). In an experiment carried out by Kalinowska et al. [32] on kids fattened to a body mass of 16 kg, the cholesterol content in the longest spinae muscle was 61.31 mg in 100 g, while in the study by Patli et al. [33], the content of cholesterol in the muscle tissue ranged from 70 to 120 mg/100 g. Madruga et al. [27] showed the effects of the age of slaughter and castration on the content of cholesterol in the muscles of the studied goat kids. In the meat of goats castrated and slaughtered at different ages, i.e., 175 days, 220 days, 265 days and 310 days, the highest cholesterol content was found in the muscles of the oldest animals—74 mg in 100 g, and the lowest cholesterol content in the group of kids slaughtered at 220 days—51.8 mg in 100 g. The meat of the castrated animals contained 62.5 mg in 100 g and non-castrated, 58 mg of cholesterol per 100 g. In addition, the meat from castrated animals had a higher fat content. Despite the lower protein content, Carpathian goat meat had a higher total collagen content compared to meat from the Saanen goats. A similar collagen content of 0.40–0.45% was noted by Marichal et al. [34] in the meat from kids of the Canary Caprine Group Breed. Stanisic et al. [35] indicated that in the meat of crossbred kid goats (Balkan × Saanen goat), along with the increase in the share of Saanen genes, the level of fat and collagen increased, while the level of protein decreased. In addition, along with the increase in the share of the Saanen genes, the shear force and thermal loss of the *m. longissimus dorsi* muscle increased during baking. In the meat of the Balkan goats, 0.34% of collagen was found. It should be borne in mind that a high collagen content in muscle tissue contributes to a decrease in digestibility and, thus, results in less tenderness and lower nutritional value of the meat. In addition, collagen is a defective protein due to the lack of tryptophan and the low content of sulphur as well as aromatic amino acids [36].

However, this did not cause any deterioration in the shear force texture parameters of the Carpathian goat meat; to the contrary, the meat was characterised by lower cutting force as well as hardness (statistically significantly lower) and chewiness, i.e., it was a tender meat (Table 2). 

**Figure 1:**
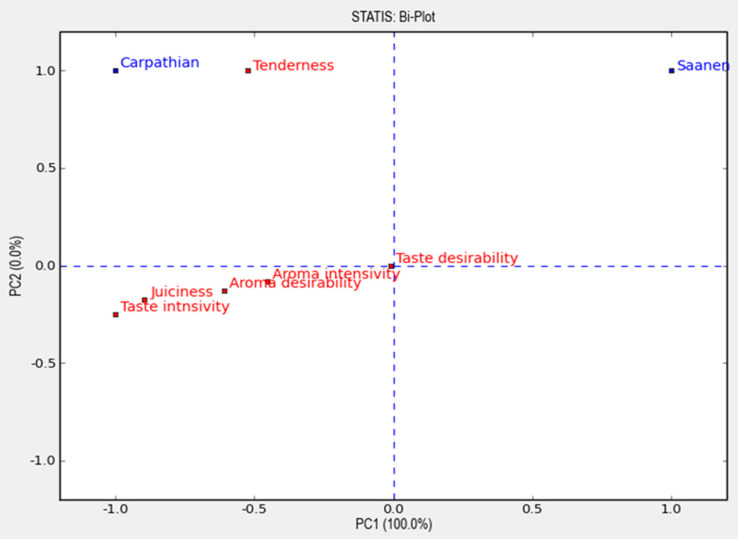
Biplot.

For sensory evaluation, roasted meat from the Carpathian goats obtained for almost all parameters (except taste desirability) higher marks compared to the meat of the Saanen goats. Biplot analysis showed that overall acceptance of meat from Carpathian goats was higher compared to Saanen goats (Figure 1). With PC1 (100% of total variance) they were negatively loaded with taste intensity, juiciness and aroma desirability. Mean/STD plots showed differences only for juiciness, while there was no statistical differences for assessors. 

The meat cutting force for the Carpathian and the Saanen goats did not differ significantly (*p* > 0.05). According to Santos et al. (2008), the cutting force of meat from Portuguese goats was 7.7 kg/cm, while for meat from kids of the Canary Caprine Group breed, this value ranged from 43.67 N (10 kg live weight at slaughter) to 68.42 N (25 kg live weight at slaughter) [34]. Stanišić et al. [35] showed that the goat meat of a native Serbian breed, the Balkan goat, was characterised by a lower cutting force and thermal losses compared to the meat of a crossbred goat with the increase in the share of Saanen genes in the genotype. During sensory evaluation, roasted Balkan goat meat was characterised by a more favourable smell; however, it had a poorer taste, juiciness and tenderness compared to the meat of goat kid crossbreeds. The juiciness of meat is directly associated with the content of intramuscular fat, water and collagen [35], but water, which remains in the product after cooking, has the greatest impact on juiciness during the consumption of meat [37].

In Table 3, the colour parameters of raw meat and thermal losses during cooking are presented. The colour parameters of kid goat meat of both breeds did not differ statistically (*p* > 0.05). The value of parameter L* of the goat meat ranged from 41.39 to 42.61, parameter a* from 17.51 to 17.74 and parameter b* from 10.87 to 11.58. Santos et al. [38], analysing the colour parameters of goat meat of Portuguese breeds, noted similar values: L* = 47.3, a* = 17.0, b* = 5.2. Bonvillani et al. [39], assessing the colour parameters of Argentine goats, also obtained similar values: L* = 42.7, a* = 10.62, b* = 15.47, respectively, while Kaić et al. [40], in the case of Boer kids meat, noted the following values: L* = 46.18, a* = 16.64 and b* = 7.40, respectively. These results indicate that the kid goat meat belongs to a dark red meat. Carpathian goat meat was statistically significantly lower in heat losses (35.31%) when roasting compared to Saanen goat meat (37.14%). According to Bonvillani et al. [39], thermal losses in the meat of Argentine goats were 24.76%, while in the meat of female 4-year-old Balkan and Serbian white goats, these values were in the range of 39.41–40.60% [41]. Liotta et al. [42] found the impact of the goat breeding system on the thermal loss of meat. Meat from male and female Messinese goats was kept exclusively outdoors and their pasture areas were characterised by the presence of *Quercussuber*; therefore, the animals also fed acorns (extensive system) had greater thermal losses (25.53%) compared to males and females fed exclusively during spontaneous pasture; in the evening, the dams were kept in the stable (semi-extensive system)—they had thermal losses of 19.60%. Warner–Bratzler shear force of the meat was 5.04 and 3.63 kg/cm^2^, respectively.

Fat is a carrier of flavour–smell compounds for meat and meat products because it has the ability to dissolve flavour and smell substances. Therefore, lean meat and meat products are less tasty and juicy compared to meat and meat products with a moderate fat content. However, fat is perceived by nutritionists as the least desirable food ingredient, which is credited with the responsibility for developing so-called lifestyle diseases (chronic environmental diseases), mainly cardiovascular. In recent years, analyses of the relationship between saturated fat intake (SFA) and cardiovascular diseases (CVD) did not show statistically significant relationships [43]. Hooper et al. [44], analysing the results of 27 major research projects, showed the existence of a weak (statistically unconfirmed) relationship between the amount of consumed fat and the level of cholesterol in blood serum and the incidence of heart disease. The fatty acid profile seems to be more important. According to nutritional recommendations, the correct ratio between PUFA—MUFA should be within the range of 0.4–1, while the ratio of n-6 PUFA to n-3 PUFA should be 2.5–8.0. In Table 4, the profile of fatty acids regarding intramuscular fat of the analysed breeds, is presented. It was reported [45] that *m. semimembranosus* is considered as a muscle with low fat content (compared to *longissimus lumborum*, *psoas major* and *gluteus medius*). The fat of Carpathian goat breeds was characterised by a higher content of monounsaturated acids, mainly C 18:1n:9 acid and a more favourable (lower) saturation index, S/P. Desaturases play a key role in the composition of the fatty acid profile in adipose tissue and animal products such as meat and milk Stearoyl-CoA desaturase is an enzyme from the class of oxidoreductase, which catalyses the formation of a fatty acid double bond between C9 and C10 [46]. Sikora et al. [47] showed that the activity of D9-desaturase in intramuscular fat was significantly greater in kids aged 90 and 180 days than in younger kids aged 60 days. The ratio of PUFA—MUFA of the analysed intramuscular fat of goats was 0.27–0.33 and, thus, below the recommended range of 0.4–1. A more favourable ratio of n-6 PUFA to n-3 PUFA was found in the fat of the Saanen goats. Many authors, when analysing intramuscular fat of the goat, found a more favourable ratio of these acids, e.g., Peña et al. [48]—2.58–4.04, Peña et al. [49]—3.19–3.29, Lopes et al. [50]—1.70–3.94. 

Two indices are of particular importance: TI (thrombogenicity index) and AI (atherogenicity index), which provide information as to what extent a particular element of a diet containing fatty acids affects the deepening of ischemic myocardial changes and, as a consequence, increases the risk of coronary artery disease. The higher values of these indicators, the greater the likelihood of blood clots and atherosclerosis [57]. In terms of these two indices, the intramuscular fat of Carpathian goat breeds is more favourable. The fat of ruminants is a rich source of CLA. In the analysed goat fat, 0.62–0.63% CLA was found, while Peña et al. [49] noted 0.97–1.0% CLA in fat of Criollo Cordobe goats and 0.81–0.85% in fat of Anglo Nubian kids. Horoszewicz and Pieniak-Lendzion [29] found that better parameters of the fatty acid profile were in meat obtained from animals slaughtered on the 150th day of life compared to goats slaughtered at the age of 90 days. Pieniak-Lendzion et al. [58] showed, however, that the meat of older goats (180 days) had a better sensory rating compared to the 90-day-old goats. The maintenance and nutrition system also affects the quality of the meat. In the meat of extensively kept Messinese goat breeds, Liotta et al. [42] found (pasture was characterised by the presence of *Quercus suber*; the animals were also fed acorn) more UFA, especially C18:1n9 and C18 2n6 acids compared to those exclusively fed on spontaneous pasture. Whereas Lopes et al. [50] showed that goats fed ad libitum, had a better quality of meat due to the lower amounts of soluble collagen and a more favourable fatty acid profile for human health with greater concentrations of oleic acids, unsaturated fatty acids and CLAs. Peña et al. [48], Madruga et al. [59] and Lopes et al. [50] demonstrated that the different goat genotypes displayed small differences in the quality of their meat and fatty acid profiles. The PUFA/SFA ratios were from 0.23 ± 0.04 (Carpathian) to 0.27 ± 0.15 (Saanen), below those suggested by Wood et al. [53], who recommended values above 0.4 to prevent illness associated with the consumption of fats. Chen and Liu [60] reported, based on the available literature, that, for meat, this ratio was 0.165 to 1.32, however, there were a lack of data for goat meat. The highest value of this ratio in the manuscript mentioned above was found for lamb meat. Ivanović et al. [61], analysing meat from Balkan, Alpine and Saanen goats, found those ratios to be most favourable in Saanen goats in contrast to Balkan goats (0.071 and 0.089, respectively). It must be mentioned that the goats were 4 years old. Sikora and Borys [62] showed a decrease in this indicator with the age of animals: 0.60 on day 60, 0.30 on day 90 and 0.196 on day 180 of slaughter, as the PUFA content dropped from 21.34 on day 60 to 8.20 g/100 g of fat on day 180. The meats present a naturally polyunsaturated—saturated ratio of around 0.1 [63], which implies an unbalanced consumption of desirable fatty acids (C18:3, C18:2 and C18:1). MUFA and PUFA acids (n-3 and n-6) have a positive effect on human health because they possess anti-atherosclerotic properties. In contrast, saturated fatty acids C12:0, C14:0 and C16:0 exhibit atherogenic effects (they cause an increase in the concentration of total cholesterol and LDL fractions). Furthermore, C14:0 and C16:0 as well as C18: 0 acids have a thrombogenic effect (they stimulate platelet aggregation). Therefore, the higher the values of the AI, TI, S/P and NV indices and the lower h/H, the lower the health-promoting quality of meat [64]. The above-mentioned indices in our study were not significant except S/P, but all of the meat from the Carpathian goats was characterized by more favourable values compared to the Saanen goats. 

Fatty acids affect the sensory characteristics of meat; mainly softness, palatability and smell [65]. The meat softness index is defined as the ratio between (C16:1 + C18:1) and (C16: 0 + C18:0) [54]. The meat of Carpathian kid goats was characterised by a more favourable softness index and nutritional value of lipids. The sum of α-linolenic acid, DHA and EPA, referred to as consumer index (CI), should not constitute more than 3% of the sum of all intramuscular fat fatty acids [53]. In the goat meat of both breeds, the value of this index ranged from 0.93 to 1.30. The fatty acid transformation forms substances which directly affect the smell and taste of goat meat. The strong smell of goat meat is due to 4-ethylocatanoic fatty acids. This acid was detected in goat meat, lamb and mutton, as well as in cheese made from milk of these species [66]. Todaro et al. [67] claim that in addition to fatty acids, taste and aroma are also affected by other compounds: hydrocarbons, aldehydes, ketones, alcohols, furans, thiophenes, pyrrols, pyrazines, oxazoles, thiazoles and sulphurous compounds.

The nutritional and biological value of meat depends not only on protein content but mainly on amino acid composition (Table 5). The biological value of goat meat is primarily related to such essential amino acids as lysine, histidine and methionine, necessary for normal growth and development, as well as tryptophan and phenylalanine, regulating the functions of the central nervous system [31]. In the goat meat of both breeds, there were no differences between the total content of exogenous and endogenous amino acids. The EAA/NEAA ratio in the meat of the analysed breeds was similar to the results obtained by Brzostowski et al. [31]. Webb et al. [36] noted a higher EAA/NEAA ratio. Carpathian goat meat was statistically significantly higher in phenylalanine, histidine, proline, alanine and tyrosine content. According to Brzostowski et al. [31], the genotype of the kids had a significant effect on the concentrations of amino acids in meat protein. The protein of meat from crossbred kids (French Alpine × Boer), in comparison to the protein of meat from French Alpine kids, contained highly significantly more EAAs such as threonine, valine, methionine, leucine and lysine, and fewer NEAAs, such as glutaminic acid, glycine and alanine. It follows that the proportions and ratios of EAAs and NEAAs in the protein of meat from crossbreeds were more desirable than in the protein of meat from pure breeds (*p* ≤ 0.05). An important quality of meat is the content of amino acids with glucogenic, ketogenic and glucoketogenic effects. This division was made due to metabolic changes in the body. Glucogenic amino acids are such that they can be converted into glucose by gluconeogenesis. Glucogenic amino acids include: serine, histidine, arginine, cysteine, proline, alanine, glutamic acid, glutamine, aspartic acid, asparagine and methionine. Ketogenic amino acids are those that can be converted into ketone bodies in the process of ketogenesis, the formation of ketone bodies as an alternative oxidation product of free fatty acids in the liver. This group includes leucine and lysine. Glucogenic and ketogenic amino acids are: isoleucine, threonine, phenylalanine, tyrosine and tryptophan. In the meat from Carpathian goat kids, statistically significantly more glucogenic amino acids such as histidine, proline and alanine were found, while in the meat of the Saanen goats, there was statistically significantly more serine and methionine. The differences in content of ketogenic amino acids in goat meat of both breeds were not of statistical significance.

## 4. Conclusions

Comparing the meat of the male goat kids slaughtered at 150 days of age, it was found that the meat of the Carpathian goat is characterised by a lower content of protein and cholesterol and a greater variety of ingredients and collagen in comparison to the meat of Saanen goats. Despite the higher collagen content, the goat meat of Carpathian breeds is characterised by a lower tensile strength and less hardness and chewiness, it is a more delicate meat. Carpathian goat fat is characterised by a higher content of monounsaturated acids, mainly C 18:1n:9 and a more favourable (lower) value of the saturation index, S/P. In the goat meat of both breeds, no differences were found between the total content of exogenous and endogenous amino acids. The EAA/NEAA ratio in the meat of the analysed breeds was 0.88–0.89. However, meat from the Carpathian goats are characterised by statistically significantly higher content of phenylalanine, histidine, proline, alanine and tyrosine in comparison with meat from the Saanen goats. The obtained results confirm the high quality of the meat of the local Carpathian breed in comparison to the Saanen breed.

## Figures and Tables

**Table 1:** Chemical composition of goat kid meat.

Parameters	Breed of Goat Kids		*p*	Significance of Differences
	Carpathian	Saanen		
Water (%)	76.92 ± 1.245	76.32 ± 1.42	0.1914	NS
Total solids (%)	23.08 ± 1.281	23.68 ± 1.445	0.1914	NS
Protein (%)	19.05 ± 1.021	20.198 ± 1.016	0.0018	XX
Fat (%)	2.89 ± 0.716	2.31 ± 0.632	0.0140	X
Ash (%)	1.07 ± 0.057	1.10 ± 0.051	0.0728	NS
Carbohydrates (%)	0.065 ± 0.038	0.073 ± 0.035	0.5006	NS
Cholesterol (mg/100 g)	55.08 ± 0.842	56.79 ± 0.659	0.0000	XX
Total collagen (%)	0.435 ± 0.047	0.373 ± 0.048	0.0005	XX

Explanations: *p*: probability; NS: not significant; X: *p* < 0.05; XX: *p* < 0.01.

**Table 2:** Texture parameters and shear force value (±SE) of the goat kid meat.

Parameters	Breed of Goat Kids		*p*	Significance of Differences
	Carpathian	Saanen		
Shear force (N)	42.46 ± 9.66	47.4 ± 9.08	0.122451	NS
Texture profile:				
Hardness (N)	65.11 ± 11.95	75.12 ± 15.77	0.0391	X
Springiness	0.42 ± 0.04	0.45 ± 0.05	0.0669	NS
Cohesiveness	0.46 ± 0.05	0.44 ± 0.03	0.0616	NS
Chewiness (N)	12.79 ± 3.32	14.57 ± 2.97	0.0984	NS
Resilience	0.18 ± 0.04	0.15 ± 0.01	0.0058	XX

Explanations: *p*-probability; NS-not significant; X-*p* < 0.05; XX-*p* < 0.01.

**Table 3:** Selected quality characteristics of the analysed meat breeds.

Parameters	Breed of Goat Kids		*p*	Significance of Differences
	Carpathian	Saanen		
Colour				
Lightness (L*)	41.39 ± 2.96	42.61 ± 3.52	0.2695	NS
Redness (a*)	17.74 ± 1.26	17.51 ± 2.54	0.7274	NS
Yellowness b*	10.87 ± 1.49	11.58 ± 2.44	0.2965	NS
Cooking loss (%)	35.31 ± 1.823	37.14 ± 2.313	0.0124	X

Explanations: *p*: probability; NS: not significant; X: *p* < 0.05; XX: *p* < 0.01.

**Table 4:** Fatty acid composition in intramuscular fat from male kid goats (%).

Fatty Acids	Breed of Goat Kids		*p*	Significance of Differences
	Carpathian	Saanen		
C 10:0	0.12 ± 0.03	0.10 ± 0.3	0.0617	NS
C 12:0	0.18 ± 0.03	0.18 ± 0.06	0.9672	NS
C 14:0	2.40 ± 0.64	2.14 ± 0.69	0.3047	NS
C 14:1	0.11 ± 0.05	0.13 ± 0.096	0.4256	NS
C 15:0	0.73 ± 0.10	0.87 ± 0.14	0.0011	XX
C 16:0	23.89 ± 0.69	24.56 ± 2.29	0.2398	NS
C 16:1n:9	1.02 ± 0.15	1.46 ± 0.72	0.0147	X
C 16:1n:7	1.78 ± 0.38	1.50 ± 0.15	0.0064	X
C 17:0	1.27 ± 0.10	1.37 ± 0.16	0.0291	X
C 17:1	0.99 ± 0.12	0.87 ± 0.14	0.0108	X
C 18:0	18.37 ± 1.87	20.21 ± 2.34	0.0137	X
C 18:1n:9	35.03 ± 1.76	32.11 ± 2.78	0.0006	XX
C 18:1n:7	2.48 ± 0.13	2.44 ± 0.30	0.6250	NS
C 18:2n:6 (LA)	6.79 ± 0.60	6.83 ± 2.60	0.9477	NS
C 18:3n:6 (GLA)	0.14 ± 0.01	0.17 ± 0.03	0.0002	XX
C 18:3n:3(ALA)	0.55 ± 0.03	0.67 ± 0.17	0.0041	X
C 18:2c9t11 (CLA)	0.62 ± 0.28	0.63 ± 0.27	0.8877	NS
C 20:0	0.19 ± 0.02	0.23 ± 0.07	0.0343	X
C 20:1	0.07 ± 0.01	0.07 ± 0.02	0.8872	NS
C 20:2	0.06 ± 0.03	0.08 ± 0.03	0.0806	NS
C 20:3n:6 (DGLA)	0.13 ± 0.04	0.13 ± 0.08	0.8473	NS
C 20:4n:6 (AA)	1.86 ± 0.66	1.83 ± 1.32	0.9389	NS
C 20:4n:3	0.02 ± 0.01	0.01 ± 0.004	0.0307	X
C 20:5n:3 (EPA)	0.11 ± 0.02	0.15 ± 0.09	0.0731	NS
C 22:4n:6	0.21 ± 0.05	0.17 ± 0.09	0.1121	NS
C 22:5n:6	0.033 ± 0.009	0.026 ± 0.013	0.0437	X
C 22:5n:3 (DPA)	0.45 ± 0.13	0.53 ± 0.28	0.3047	NS
C 22:6n:3 (DHA)	0.13 ± 0.06	0.31 ± 0.27	0.0106	X
Other	0.30 ± 0.06	0.20 ± 0.11	0.0020	X
SFA	47.11 ± 0.56	49.32 ± 4.01	0.0269	X
UFA	52.59 ± 0.58	50.68 ± 3.89	0.0297	X
MUFA	41.49 ± 2.17	38.35 ± 2.18	0.0001	XX
PUFA	11.09 ± 1.88	12.33 ± 5.96	0.4873	NS
n6	9.17 ± 1.36	9.17 ± 4.09	0.9988	NS
n3	1.26 ± 0.24	1.67 ± 0.75	0.0310	X
n6/n3	7.36 ± 0.32	5.52 ± 0.20	0.0000	XX
UFA/SFA	1.12 ± 0.03	1.04 ± 0.16	0.0481	X
PUFA/SFA	0.23 ± 0.04	0.27 ± 0.15	0.0166	X
PUFA/MUFA	0.27 ± 0.06	0.33 ± 0.18	0.2204	NS
DFA	70.95 ± 1.32	70.68 ± 3.01	0.7299	NS
OFA	28.74 ± 1.33	29.11 ± 3.09	0.6459	NS
DFA/OFA	2.48 ± 0.16	2.46 ± 0.35	0.8892	NS
h/H	1.708 ± 0.091	1.627 ± 0.267	0.1054	NS
DI	0.51 ± 0.04	0.44 ± 0.009	0.0000	XX
AI	0.51 ± 0.02	0.55 ± 0.09	0.0867	NS
TI	1.52 ± 0.04	1.65 ± 0.33	0.1191	NS
S/P	0.85 ± 0.02	0.94 ± 0.15	0.0166	X
A-SFA	26.43 ± 1.28	26.88 ± 2.99	0.5655	NS
T-SFA	44.51 ± 0.59	46.69 ± 3.76	0.0201	X
CI	0.93 ± 0.11	1.30 ± 0.49	0.0035	XX
Meat softness index	0.97 ± 0.078	0.84 ± 0.021	0.019	X
NV	0.63 ± 0.031	0.69 ± 0.087	0.131	NS

Explanations: *p*-probability; NS: not significant; X: *p* < 0.05; XX: *p* < 0.01. LA: linoleic acid; GLA: γ linolenic acid; ALA: α-linolenic acid; CLA: conjugated linoleic acid; DGLA: dihomo-γ-linolenic acid; AA: arachidonic acid; EPA: eicosapentaenoic fatty acids, DHA: docosahexaenoic fatty acids; SFA: saturated fatty acids, MUFA: monounsaturated fatty acids, PUFA: polyunsaturated fatty acids, UFA: unsaturated fatty acids, DFA: hypocholesterolemic fatty acids, OFA: hypercholesterolemic fatty acids; S/P: saturation index–SFA/UFA (C14:0 + C16:0 + C18:0)/(MUFA + PUFA) [50]; AI: atherogenic index—(C12:0 + 4 × C14:0 + C16:0) [(MUFA + ΣSPUFA (n6) + (n3)] [50];. TI: thrombogenic index—(C14:0 + C16:0 + C18:0) ÷ 0.5 × MUFA + 0.5 × n6PUFA + 3 × n3PUFA + n3PUFA) ÷ n6PUFA) [51]; A-SFA—(C12:0 + C14:0 + C16:0); T-SFA—(C14:0 + C16:0 + C18:0); Δ9-desaturase index—(C14:1n9 + C16:1n9 + C18:1n9) ÷ (C14:1n9 + C18:1n9 + C18:1n9 + C14:0 + C16:0 + C18:0) [52]; CI: consumer index—(C18:3 + C20:5 + C22:6) [53]; Meat softness index—(C16: 1 + C18: 1) ÷ (C16:0 + C18: 0) [54]; NV: nutritional value of lipids—(C12:0 + C14:0 + C16:0) ÷ (C18:1 c9 + C18:2 n-6) [55]; h/H: ratio of hypo- and hypercholesterolemic acids—(C18:1 c9 + C18:2 n-6 + C18:3 n-6 + C18:3 n-3 + C20:2 n-6 + C20:3 n-6 + C20:4 n-6 + C20:3 n-3 + C20:4 n-3 + C20:5 n-3 + C22:4 n-6 + C22:5 n-6 + C22:5 n-3 + C22:6 n-3) ÷ (C12:0 + C14:0 + C16:0) [56].

**Table 5:** Amino acid profile of protein in meat from male kid goats (%).

Amino Acids	Breed of Goat Kids		*p*	Significance of Differences
	Carpathian	Saanen		
Threonine	5.38 ± 0.40	5.54 ± 0.36	0.2173	NS
Valine	5.92 ± 0.10	5.96 ± 0.26	0.6583	NS
Methionine	3.07 ± 0.06	3.16 ± 0.13	0.0124	X
Isoleucine	5.37 ± 0.26	5.26 ± 0.13	0.1163	NS
Leucine	9.38 ± 0.48	9.38 ± 0.28	0.9988	NS
Phenylalanine	4.88 ± 0.46	4.55 ± 0.39	0.0283	X
Histidine	3.26 ± 0.21	2.99 ± 0.27	0.0021	XX
Lysine	9.67 ± 0.70	9.98 ± 0.63	0.1620	NS
*Total EAAs*	46.94 ± 1.41	46.82 ± 0.55	0.7491	NS
Aspartic acid	9.33 ± 0.21	9.61 ± 0.57	0.0565	NS
Serine	3.73 ± 0.15	3.89 ± 0.21	0.0127	X
Glutamic acid	14.97 ± 0.67	15.07 ± 0.53	0.6137	NS
Proline	4.19 ± 0.23	3.85 ± 0.34	0.0010	XX
Glycine	4.11 ± 0.88	4.48 ± 0.39	0.1173	NS
Alanine	6.03 ± 0.25	5.82 ± 0.27	0.0212	X
Tyrosine	4.01 ± 0.20	3.76 ± 0.43	0.0323	X
Arginine	6.70 ± 0.15	6.71 ± 0.19	0.9717	NS
*Total NEAAs*	53.06 ± 1.41	53.18 ± 0.55	0.7353	NS
EAAs/NEAAs	0.89 ± 0.05	0.88 ± 0.02	0.6848	NS

Explanations: *p*: probability; NS: not significant; X: *p* < 0.05; XX: *p* < 0.01.

## Data Availability

Data available on request due to restrictions eg privacy or ethical.
